# Extracellular vesicles as a novel photosensitive drug delivery system for enhanced photodynamic therapy

**DOI:** 10.3389/fbioe.2022.1032318

**Published:** 2022-09-27

**Authors:** Lingjun Tong, Sitong Zhang, Rong Huang, Huaxi Yi, Jiong-Wei Wang

**Affiliations:** ^1^ Medical Science and Technology Innovation Center, Shandong First Medical University and Shandong Academy of Medical Sciences, Jinan, China; ^2^ Department of Surgery, Yong Loo Lin School of Medicine, National University of Singapore, Singapore, Singapore; ^3^ Nanomedicine Translational Research Programme, Centre for NanoMedicine, Yong Loo Lin School of Medicine, National University of Singapore, Singapore, Singapore; ^4^ College of Food Science and Engineering, Ocean University of China, Qingdao, China; ^5^ Cardiovascular Research Institute, National University Heart Centre Singapore, Singapore, Singapore; ^6^ Department of Physiology, Yong Loo Lin School of Medicine, National University of Singapore, Singapore, Singapore

**Keywords:** extracellular vesicle, nanoparticle, photosensitizer, drug delivery system, photodynamic therapy

## Abstract

Photodynamic therapy (PDT) is a promising non-invasive therapeutic approach that utilizes photosensitizers (PSs) to generate highly reactive oxygen species (ROS), including singlet oxygen, for removal of targeted cells. PDT has been proven efficacious for the treatment of several diseases, including cancer, cardiovascular disease, inflammatory bowel disease, and diabetic ocular disease. However, the therapeutic efficacy of PDT is limited and often accompanied by side effects, largely due to non-specific delivery of PSs beyond the desired lesion site. Over the past decade, despite various nanoparticular drug delivery systems developed have markedly improved the treatment efficacy while reducing the off-target effects of PSs, concerns over the safety and toxicity of synthetic nanomaterials following intravenous administration are raised. Extracellular vesicles (EVs), a type of nanoparticle released from cells, are emerging as a natural drug delivery system for PSs in light of EV’s potentially low immunogenicity and biocompatibility compared with other nanoparticles. This review aims to provide an overview of the research progress in PS delivery systems and propose EVs as an alternative PS delivery system for PDT. Moreover, the challenges and future perspectives of EVs for PS delivery are discussed.

## Introduction

Photodynamic therapy (PDT), a non-invasive therapeutic approach employing light irradiation and photosensitizers (PSs) to remove unwanted cells, has been proven efficacious for the treatment of several diseases, including cancers (Zheng et al., 2020; Zou et al., 2021), cardiovascular disease (Kim et al., 2013), inflammatory bowel disease (Rong et al., 2021), and diabetic ocular disease (van Rijssen et al., 2021). Compared with traditional treatment methods such as surgery, radiotherapy, and chemotherapy, PDT exhibited efficient therapeutic action and low systemic toxicity (Feng et al., 2020; Gunaydin et al., 2021). PDT mainly relies on the light-excited PSs that generate robust reactive oxygen species (ROS), such as singlet oxygen, hydrogen peroxide, and superoxide radicals, through the light source (Callaghan and Senge, 2018). These ROS confer high reactivity towards cellular membrane, nucleic acids, or peptides, thereby causing cell damage and triggering apoptosis (Zhang et al., 2015). Although PDT has been used for disease treatment in patients, its broad clinical applications are impeded due to limited light penetration and high oxygen dependence of PSs (Li et al., 2018; Yang M. et al., 2019). More critically, PSs may accumulate in normal tissue, resulting in off-target effects and low PDT efficacy (Zhou et al., 2016).

Over the past decade, various nanoparticular delivery systems have been developed for PSs, including liposomes, aggregation-induced emission (AIE) luminogen hybrid nanovesicles, and polymeric nanoparticles (Zhu et al., 2020; Pan et al., 2021). These drug delivery systems could increase the targeting of PSs and enhance PDT therapeutic effects likely due to the enhanced permeability and retention (EPR) effect *via* which nanoparticles of size <500 nm go through the leaky vasculature and accumulate within tumors and other inflamed tissue sites (Voon et al., 2014; Li and Wang, 2021; Shen et al., 2022). In addition, nanoparticular delivery systems can deliver PSs to the diseases site through active targeting by conjugating specific ligands or other modifications on the surface of nanoparticles (Kumar et al., 2008). However, the safety and toxicity of PSs delivery systems remain a major challenge in their clinical applications (Zheng et al., 2020). Therefore, there is an urgent demand to develop viable PS delivery systems for more targeted and safer PDT.

Extracellular vesicles (EVs) are natural and lipid bilayer-delimited nanoparticles secreted from various types of cells, with better biocompatibility, lower immunogenicity, higher efficiency, and better EPR effect compared with synthetic nanoparticles (Zhu et al., 2020; Escude Martinez de Castilla et al., 2021). These outstanding properties make them promising candidates for drug delivery, which have attracted great interest from researchers in recent years. Previous studies have indicated that EVs own abundant tetraspanins (CD9, CD81, CD63), which could promote drug delivery through membrane fusion between EVs and target cells (Cheng et al., 2018). Furthermore, CD47, a “Don’t Eat Me” signaling protein, is expressed on most EVs, especially those derived from tumor cells (Kamerkar et al., 2017). It enables EVs to escape phagocytosis by immune cells for drug delivery. A recent study reported that tumor cell-derived EVs exhibited better immune escape and tumor targeting (Qiao et al., 2020). In addition, EVs can be modified using multiple approaches, such as incubation, sonication, and extrusion (Xu et al., 2020). Ligands used in EV modification are discussed in more depth in a recent review by Escude Martinez de Castilla et al. (2021). We documented that the most common types of targeting ligands used in EVs for drug delivery are small peptides (38%), transmembrane proteins (34%), and antibody fragments (25%).

In this review, we first discuss the advantages and limitations of conventional nanoparticles for PS delivery, then we focus on potential applications of EVs as an alternative delivery system for PS administration in PDT. Our literature analysis suggests that EVs may extend PDT beyond cancer treatment to other diseases, such as cardiovascular disease, inflammatory bowel disease, and diabetic retinopathy. Further research on EV delivery of PSs for PDT in non-cancer disease treatment is warranted.

## Photosensitizers and nanoparticular delivery systems

### Photosensitizers and application

PSs have been evolving from the first generation to the third generation (Moghassemi et al., 2021). The first-generation PSs were hematoporphyrin derivative (HpD) or porfimer sodium, such as porphyrin monomers, dimers, and oligomers (Zheng et al., 2020). The main limitations include low tissue selectivity, poor light absorption and prolonged skin photosensitivity. The second-generation PSs can be activated by specific wavelengths, usually in the range of 650–800 nm (Mokwena et al., 2018). Compared to the first-generation PSs, the second-generation PSs consist of derivatives of porphyrins, chlorins phthalocyanines, and naphthalocyanines, such as hypericin, chlorin e6 (Ce6), zinc phthalocyanine (ZnPc), and Al (III) phthalocyanine chloride tetrasulfonic acid (AlPcS4). These PSs exhibited higher yields of singlet oxygen, better tissue selectivity, and fewer side effects (Simoes et al., 2020). In order to improve the selective affinity, the third-generation PSs have been developed by modifying second-generation PSs with peptides or antibodies and the utilization of nanoparticles (Derycke and de Witte, 2004; Plenagl et al., 2019).

To date, PSs have been used to treat a variety of diseases, including cancer and non-malignant diseases (Abrahamse and Hamblin, 2016). For instance, HpD is the first FDA approved PS for cancer therapy in the late 1970s (Moghassemi et al., 2021). Since then, several PSs, such as 5-aminolevulinic acid (5-ALA) and temoporfin, have been approved for cancer therapy (Fotinos et al., 2006). Notably, with the development of endoscopy and fiberoptic technologies, PSs (e.g., 5-ALA) have been applied to treat gastrointestinal diseases, such as ulcerative colitis (Rong et al., 2021). In fact, Reinhard et al. (2015) reported a liposomal formulation of meta-tetra (hydroxyphenyl) chlorin that alleviated colitis and prevented colitis-associated carcinogenesis in mice. Moreover, PDT was proposed as a therapeutic strategy for atherosclerosis (Wennink et al., 2017). For instance, Waksman et al. (2006) reported that a photosensitizer, MV0611, reduced vascular intimal proliferation without suppressing re-endothelialisation in a porcine model of restenosis. In diabetic retinopathy, PDT seems to restore neuroretinal anatomy and potentially slow down disease progression (van Rijssen et al., 2021). Overall, despite most studies of PSs focusing on cancer therapy, PDT may be applied for the treatment of some non-malignant diseases.

### Application of nanoparticles for photosensitizer delivery

Nanoparticles as carriers for drug delivery present many outstanding properties, including size, charge, porosity, stability, permeability, hydrophilicity, and large surface area-to-volume ratio (Nkune and Abrahamse, 2021; Tu and Yu, 2022). They can accumulate in disease areas to improve treatment specificity while mitigating undesirable side effects (Naidoo et al., 2018; Nkune and Abrahamse, 2021). Target delivery is mainly divided into two ways: 1) non-specific passive target delivery achieved by size and charge design of nanoparticles to generate EPR effect and 2) specifically active target delivery by means of surface ligand modification for specific receptors (Sanna and Sechi, 2020; Tang et al., 2021). Through them, active uptake and absorption in disease sites can be promoted. Notably, the different pH, target expression, enzyme environments, microvascular structures, and intracellular reduction environments between solid tumors and healthy tissues provide natural convenience for nanoparticles to be enriched and drugs can be released in tumors. For instance, Accurins can target the extracellular domain of prostate-specific membrane antigen (PSMA) to tumors at the tissue, cellular, and molecular levels through grafting of a ligand (Hrkach et al., 2012). The targeting effect makes application of nanoparticles in cancer therapy extremely attractive (Rosenblum et al., 2018; Yu et al., 2020). Currently, nanoparticles have already been incorporated and tested in mainstream cancer therapy strategies, such as chemotherapy and immunotherapy for hepatocellular carcinoma (Muhamad et al., 2018; Riley et al., 2019).

Since nanoparticles are able to protect PSs from aggregation in aqueous environment and deliver PSs to the diseases site, they have been extensively explored for PS delivery in PDT (Kwiatkowski et al., 2018; Alsaab et al., 2020; Hou et al., 2020). As shown in Table 1, various types of nanoparticles based on organic and inorganic materials were used for PS delivery in cancer therapy. Viral and lipid-based nanoparticles were also reported for PS delivery (Namiki et al., 2011; Lin et al., 2021). Liposomes have shown great potential for loading hydrophobic PSs (Moghassemi et al., 2021), while gold nanoparticles can be conjugated with water soluble ionic PSs such as purpurin-18-N-methyl-D-glucamine (Lkhagvadulam et al., 2013). Apart from cancer therapy, nanoparticle-based PSs were also explored for cardiovascular disease treatment. For example, upconversion fluorescent nanoparticles containing Ce6 promoted cholesterol efflux by inducing autophagy, indicating a potential capability of attenuating atherosclerotic plaque progression (Han et al., 2017). In addition, macrophage-derived nanoparticles (MacTNP) conjugated with Ce6-hyaluronic acid were suggested to mediate PDT of atherosclerosis with minimal side effects (Kim et al., 2013).

**TABLE 1:** Recent studies regarding the application of nanoparticles to PDT.

Type of nanoparticles	Cargo	Study objective	Result	Ref.
Doped- and undoped-TiO_2_ nanoparticles stabilized by PEG	Titanium dioxide	Cervical cancer cells (HeLa)	Significantly reduced cell survival	Shah et al. (2019)
Conjugation of gold nanoparticles (GNanoparticles)	5-aminolevulinic acid (5-ALA)	Cutaneous squamous cell carcinoma (cSCC)	Significantly suppressed cell viability and increased cell apoptosis	Chi et al. (2020)
TID nanoparticles	Doxorubicin (DOX)	Breast cancer cells	Rapidly destroyed the genetic substances and potently induced the apoptosis	Guoyun Wan et al. (2020)
P123 Pluronic^®^-based nanoparticles	Hypericin	Cervical cancer cells	Exerted effective and selective time- and dose-dependent phototoxic effects	Damke et al. (2020)
Poly-ε-caprolactone nanoparticles (PCL Nanoparticles)	IR780 and paclitaxel (PTX)	Ovarian cancer cells	Demonstrated increased tumor cell internalization	Pan et al. (2020a)

### Advantages and limitations of nanoparticular systems for PS delivery

As mentioned above, nanoparticle-based PS delivery systems for PDT have the advantages of good stability, high drug loading, enrichment at tumor sites, enhanced therapeutic efficacy, and reduced risk of healthy tissue damage (Hou et al., 2020). However, limitations still exist. Nanoparticles are mostly synthetic particles made of carbon, ceramics, metals, semiconductors, polymers, and lipids (Yohan and Chithrani, 2014). This leads to low biocompatibility, high immunogenicity, thus hindering their clinical applications (Yohan and Chithrani, 2014). According to existing publications, the application of PDT in tumor therapy dates back to the late 1970s (Kelly and Snell, 1976). Moreover, the research and clinical trial records presented in Table 2 demonstrated that PDT has shown clinical efficacy in certain cancers (e.g., malignant and pre-malignant skin cancers, barrett’s esophagus, and unresectable cholangiocarcinoma, and glioma) (Stylli et al., 2005; Kochneva et al., 2010). However, poor safety and tolerability have prevented the approval of PSs application in most types of cancers in clinical (Anderson et al., 2019). Consistently, nanoparticle-based PS delivery systems have barely been used in clinical trials. Most studies remain in the pre-clinical phase (Table 1), suggesting that the limitations of nanoparticles restrict their use in patients (Boraschi et al., 2017; Hou et al., 2018). As synthetic unnatural delivery systems that tend to drive safety and tolerability concerns, nanoparticles temporarily failed to serve as a practical strategy to promote the clinical application of PDT.

**TABLE 2:** Clinical trials of photodynamic therapy on cancers.

Disease	Clinical trial phase	PSs	Application of nanoparticles	Outcomes	NCT/Ref.
Skin cancer	Phase II	Radachlorin	N.A.	No side effects. Good tolerability	Kochneva et al. (2010)
Barrett’s esophagus	Phase II	Photofrin	N.A.	Good effect. No obvious side effects	Panjehpour et al. (2000)
Glioma	Phase II	Haemetaporphyrin derivative (HpD)	N.A.	Good effect. No obvious side effects	Stylli et al. (2005)
Brain tumor	Phase I	Photofrin	N.A.	Treatment’s safety not confirmed	NCT01682746
Bladder cancer	Phase II	Hexvix	N.A.	Toxicities exist	NCT01303991

## Extracellular vesicles as drug delivery systems

### Extracellular vesicles

**FIGURE 1:**
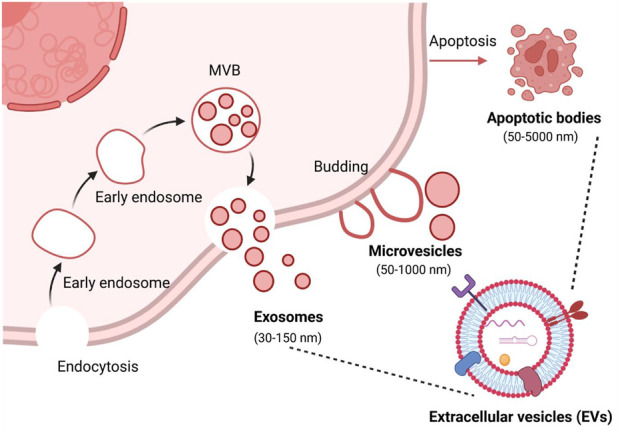
Biogenesis of the three major categories of extracellular vesicles. MVB, multivesicular bodies. Figure was prepared with BioRender^®^.

EVs are a heterogeneous group of lipid-bound nanoparticles that contain proteins, small RNAs, DNA, and lipids from parental cells (Figure 1) (Kalluri and LeBleu, 2020). EVs exist in all cells, bacteria (Tong et al., 2021b), and body fluids, such as urine (Zhu Q. et al., 2021), blood (Minghua et al., 2018), saliva (Mi et al., 2020) or milk (Tong et al., 2020; Tong et al., 2021a). They can be divided into three main populations including exosomes, microvesicles, and apoptotic bodies (Andaloussi et al., 2013). Exosomes are small vesicles with a size range of 30–150 nm in diameter, and are generated and contained intracellularly inside multivesicular bodies (MVBs). The MVBs can fuse with the plasma membrane to release intraluminal vesicles as exosomes (van Niel et al., 2018). Microvesicles range in size from 50 to 1,000 nm and are released from cells by budding directly from the membrane (Willms et al., 2018). Apoptotic bodies (50–5,000 nm) are also released from the plasma membrane, but only by cells undergoing apoptosis (Andaloussi et al., 2013). The heterogeneity among EV populations has greatly hindered their characterization at the subtype level and the study of differences between subtype functions, such as overlapping sizes and the lack of specific proteins in each subtype (Kowal et al., 2016).

Compelling evidence suggests that EVs are not only involved in pathophysiological processes, such as immune responses, cancer progression, cardiovascular diseases, and central nervous diseases, but also serve as a promising carrier for the delivery of modern therapeutic payloads, including small RNAs, chemotherapeutic agents, antisense oligonucleotides, and immunomodulators (Kalluri and LeBleu, 2020; Herrmann et al., 2021). A systemic review published by us showed that the majority of studies (66.2%) on EVs for drug delivery over the past decade have focused on cancer therapy, followed by cardiovascular diseases such as myocardial infarction and stroke (Escude Martinez de Castilla et al., 2021). Interestingly, we found that cancer cells (23.6%), stem cells (22.9%), and HEK293 (21.7%) derived EVs were most commonly used in preclinical studies (Escude Martinez de Castilla et al., 2021). This may be because researchers are trying to take advantage of the homing and immune escaping properties of EV pararenal cells, such as cancer cells and stem cells. Due to the seemingly advantages of EVs compared to conventional synthetic nanocarriers, they are considered as next-generation drug delivery platforms for PDT.

### Isolation and purification of EVs

The purity and yield of EVs are important aspects for downstream research and applications, especially drug delivery. Although the technologies currently applied in isolating EVs have shown high efficiency, the lack of EV purity and yield remains a significant concern and challenge. To date, no one standard method for isolation and purification of EVs. The main isolation methods of EVs have been well documented in some literature (Li et al., 2017; Huang et al., 2021), including differential centrifugation, density gradient centrifugation (DGC), size exclusion chromatography (SEC), immunoaffinity capture-based technology (IAC), commercial EVs isolation kits, and microfluidic technology (Table 3).

**TABLE 3:** Commonly isolation methods of EVs.

Methods	Principle	Advantage	Disadvantage
Differential centrifugation	The specific sedimentation coefficients of EVs	Easy to operate, and high yield	Time-consuming and low recovery
Density gradient centrifugation	The specific sedimentation coefficients of EVs	High purity	Time-consuming
Size exclusion chromatography	The different size of particles	High purity and recovery	Complex method
Immunoaffinity capture-based technology	The expression of specific antigens on the surface of EVs	High purity	High reagent cost, low yield
Commercial EVs isolation kits	Co-precipitation with PEG	Easy to operate	Low purity and yield
Microfluidic technology	The “size-based” and “immunoaffinity-based” principles	High purity, good portability	High reagent cost, small sample capacity

Differential centrifugation is currently widely used as the gold standard method to isolate EVs (Thery et al., 2006). This approach is easy to operate, and the yield of EVs is high, however, the limitations are time-consuming and low recovery of EVs. In addition, there is evidence that severe mechanical damage to EVs occurs if ultracentrifugation exceeds 4 h (Cvjetkovic et al., 2014). DGC is based on the specific sedimentation coefficients of EVs, which can be enriched in the range of 1.13–1.19 g/ml sucrose or iodixanol gradients. Compared with differential centrifugation, DGC significantly improves the purity and quality of isolated EVs. Although DGC is considered one of the best methods for EVs purification, some contaminants, including low/high-density lipoproteins (LDL/HDL) are still observed in EVs fractions (Sodar et al., 2016). SEC is based on the different size of particles. That is, macromolecules cannot penetrate the porous gel pores and are eluted before smaller particles. However, small nanoparticles like EVs can penetrate the gel pores and eventually be eluted by the flow phase (Monguio-Tortajada et al., 2019). SEC has been successfully used to isolate and purify EVs from various biological fluids, such as blood (Brahmer et al., 2019), urine (Oeyen et al., 2018), milk (Blans et al., 2017), and cell-conditioned media (Liu et al., 2020). In addition to enhancing EV recovery and purity, SEC-based EVs isolation methods can preserve EV structure, integrity, and biological activity (Gamez-Valero et al., 2016). A recent review suggests that SEC may be superior to other EV isolation and purification methods reported at the ISEV Virtual Conference 2020 (Sidhom et al., 2020). IAC relies on the expression of specific antigens on the surface of EVs and can be captured by incubation with magnetic beads conjugated to relative antibodies. This method can remove residual proteins and debris in the solution, resulting in high-purity EVs (Tauro et al., 2012). Notably, IAC can maintain the morphological integrity of EVs and enable the isolation of tissue-derived EVs from plasma based on EV-specific surface markers. The major limitation of this approach is that, due to the solution pH and salt reagents used during the IAC process, the biological activity of EVs may easily be affected, thereby affecting downstream experiments. Recently, commercial kits have been rapidly developed due to their simple and quick EV-isolation procedures. To date, various types of commercial kits have been developed based on the principle of co-precipitation or size differentiation, such as ExoQuick™ exosome precipitation solution, RIBO™ EVs isolation reagent, and iZON sciences. The advantages of commercial kits are that they are robust and fast, and they save samples for EV isolation, especially for subsequence miRNA identification (Ding et al., 2018). However, co-precipitation of protein complexes is unavoidable during EV isolation in most kits. Microfluidics is an emerging and promising avenue to optimize EV isolation and purification. Microfluidic systems for EV isolation are generally classified into two types, including “size-based” and “immunoaffinity-based” principles (Meng et al., 2021). Both of them can offer a route toward rapid and efficient EV separation. In addition to high purity and sensitivity, microfluidic systems can also robustly produce mimetics for drug delivery (Meng et al., 2021). However, from a device perspective, the main parts in a microfluidic system are intended for single-use applications. Considering the complexity of their fabrication, the application of microfluidic systems will be limited in biological experiments.

### Passive and active loading strategies of photosensitizers into EVs

**FIGURE 2:**
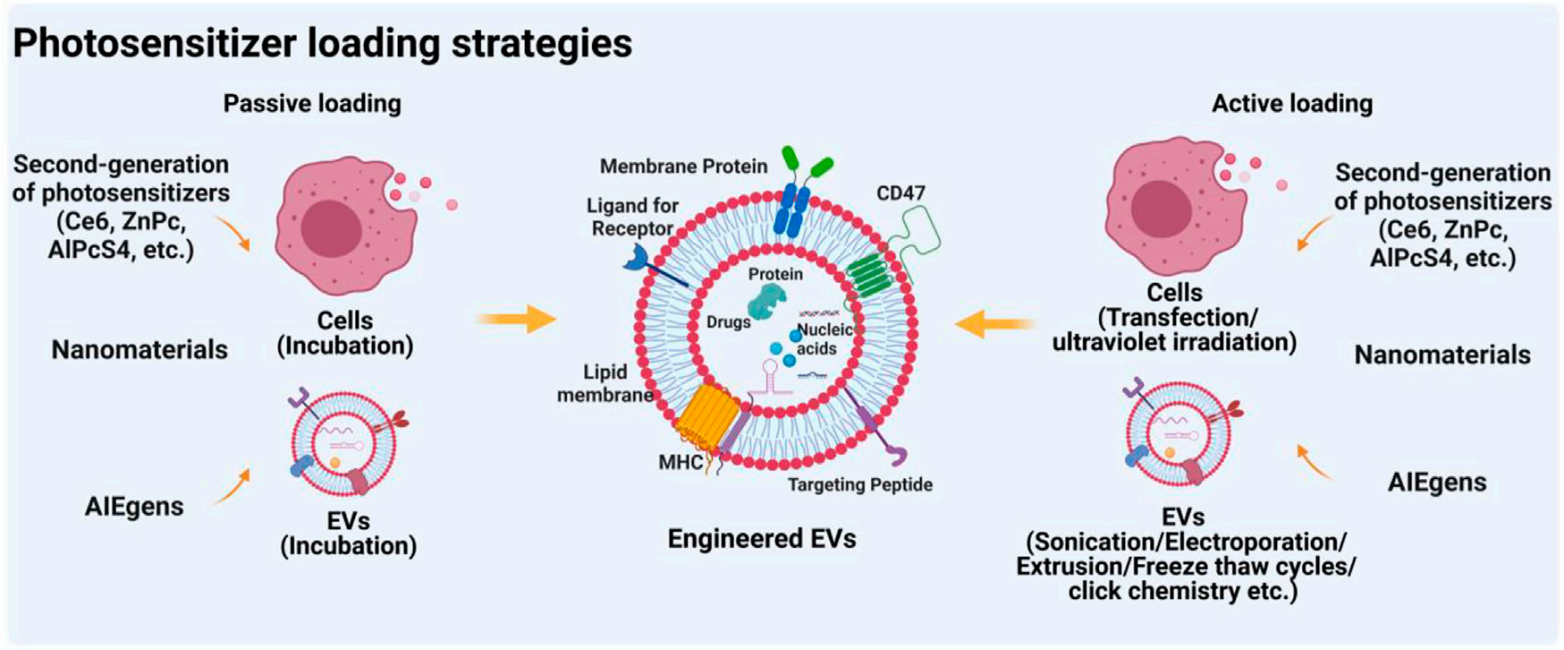
Strategies for loading photosensitizers into EVs. Figure was prepared with BioRender^®^.

Cargo loading strategies for EVs can be divided into two types: passive loading and active loading (Figure 2). Passive loading refers to the direct loading of the drug into donor cells or EVs by incubation. That is, the drug can be loaded by incubation with donor cells prior to EV isolation (Guo et al., 2019). Drugs are able to enter the cytoplasm *via* phagocytosis and participate in the developing progress of EVs, then drugs were released by fusion of drug-loading EVs with the plasma membrane. Similarly, drugs were also directly loaded into isolated EVs in the same manner (Zhu et al., 2019). The loading efficiency of this method depends on the properties of the drugs. Generally, hydrophobic molecules are often used as typical drugs for EV loading due to their easy interaction with lipids on the EV surface. For instance, curcumin and doxorubicin, two commonly used hydrophobic drugs, can be loaded into EVs for cancer therapy (Aqil et al., 2017; Wang J. et al., 2018). Interestingly, Ce6 as a promising hydrophobic photosensitizer can be loaded into EVs by incubation, thereby playing a crucial role in enhancing the PDT effect (Pan S. et al., 2020). Furthermore, emerging evidence indicated that aggregation-induced emission luminogens (AIEgens) with two positive charges can be efficiently adsorbed on the lipid bilayer membrane (Wang et al., 2018b; Zhu D. et al., 2021). Thus, AIEgens used as PSs can also be incubated with tumor-derived EVs for tumor glutamine starvation therapy and enhanced type-I photodynamic therapy (Zhu et al., 2022).

The other loading approach, active loading, refers to the loading of therapeutic drugs into donor cells or EVs through additional physical or chemical intervention (de Jong et al., 2019). Before EVs isolation, drugs could be loaded into cells *via* transfection-based methods and ultraviolet irradiation, whereas after EVs isolation, drugs can be loaded into EVs by physical (electroporation, sonication, extrusion, freeze and thaw cycles, etc) or chemical procedures (click chemistry, transfection kits, etc). Our previous systematic review has investigated that drug loading methods before EVs isolation were mainly transfection-based methods (77.9%), whereas drugs loading after EVs isolation was mainly achieved through physical procedures such as electroporation (39%), plain incubation (29.3%), and sonication (12.2%) (Escude Martinez de Castilla et al., 2021). Notably, for EVs as PSs delivery systems, the drug-loading method of incubation is a mostly-used approach (Table 3). The main reason may be that these hydrophobic PSs, such as Ce6 (Pan S. et al., 2020) and ZnPc (Huis In 't Veld et al., 2022), will be easier to bind on the surface of EVs *via* incubation. Notably, although through all these active loading methods, various drugs can be loaded into EVs, the loading efficiency is uneven. An interesting study reported by Kim et al. (2016) showed that compared with the passive loading methods, all active loading methods, especially extrusion and sonication, generate higher loading efficiencies. However, these active methods may be limited due to interference from additional physical or chemical interventions. For example, electroporation with phosphate-buffered pulses may result in the aggregation of EVs (Yan et al., 2020). The membrane structure of EVs can be damaged by extrusion or freeze and thaw cycles (Fuhrmann et al., 2015). Therefore, when we load drugs into EVs, different drug loading methods can be selected according to the physicochemical properties of drugs. Hydrophobic drugs like small molecules can be loaded through passive loading methods (Haney et al., 2015). For small RNAs, such as microRNAs, siRNAs, and lincRNAs, active loading such as electroporation may be the best approach (Liang et al., 2020). Extrusion and sonication loading methods may be more suitable for hydrophilic drugs or proteins (Yuan et al., 2017).

## EVs as a PS delivery system for photodynamic therapy

With the increasing demand for safer and more effective disease treatment strategies, novel photosensitizer delivery systems with higher targeting effects, better biocompatibility, and lower immunogenicity remain further developed and designed. EVs, as a natural and promising PSs delivery system, have the ability to escape immune cells phagocytosis and precisely target specific tissue (van den Boorn et al., 2013; Batrakova and Kim, 2015) with very low immunogenicity (van den Boorn et al., 2011; Ou et al., 2022). Moreover, these EVs can cross the blood-brain barrier and penetrate deep into structural tissue (Yang et al., 2017). To date, the development and utilization of PSs delivery systems based on EVs remain primitive. Table 4 below shows some advanced and recent EV-based PS delivery studies.

**TABLE 4:** Preclinical studies on photosensitizer-loaded EVs for PDT application.

EVs source	Photosensitizer	Loading methods	Diseases	Size (nm)	Experiments	Route of administration	Ref.
Blood	Chimeric peptide (consist of PpIX and NLS)	After isolation EVs, incubation	Breast cancer	∼114	*In vitro* tests in 4T1 and Hela cells and *in vivo* tests in 4T1 tumor-bearing mice	I.V.	Cheng et al. (2019)
Urine	amphiphilic polymer (PMA)/Au-BSA@ Chlorin e6	After EVs isolation, electroporation	Gastric cancer	∼100	*In vitro* tests with MGC-803 cells and RAW264.7 cells and *in vivo* tests with MGC-803 tumor-bearing nude mice	I.V.	Pan et al. (2020b)
4T1 mammary tumor cell	(E)-4-(2-(7-(diphenylamino)-9-ethyl-9H-carbazol-2-yl) vinyl)-1-methylpyridin-1-ium hexafluorophosphate (DCPy)	After EVs isolation, electroporation	Breast cancer	∼150	*In vitro* tests in 4T1 cells and *in vivo* tests in 4T1 breast cancer tumors	I.V.	Zhu et al. (2020)
MGC803 cells	Phthalocyanine chloride tetrasulfonic acid (AlPcS4)	Before EVs isolation, incubation with cells	Gastric cancer	∼100	*In vitro* tests with MGC803 cells and MGC803 cell spheroids and *in vivo* tests with MGC803-derived tumor xenografts mice	I.V.	Gong et al. (2021)
HEK293T cells	Rose Bengal	After isolation EVs, sonication	Hepatocellular carcinoma	30–150	*In vitro* tests in Hepa1-6 cells and *in vivo* tests in Hepa1-6 cell xenograft mice	I.V.	Du et al. (2021)
MIA-PaCa-2 cells	Chlorin e6	After isolation EVs, sonication	Melanoma	44.4 ± 14.5	*In vitro* tests in MIA-PaCa-2, RAW264.7, PBMC cells and *in vivo* tests in B16F10 engrafted BALB/c nude mice	I.V.	Jang et al. (2021)
B16F10 cells	Zinc phthalocyanine	After isolation EVs, incubation	Colorectal cancer	∼120	*In vitro* tests with D1DCs, RAW, and MC38-CFP cells and *in vivo* tests with MC38 tumor-bearing mice	I.T. or I.V.	Lara et al. (2021)
M1 macrophages	Lanthanidedoped upconversion nanoparticles	After isolation EVs, sonication	Lung cancer	119.30	*In vitro* tests with LLC or A549 cells and *in vivo* tests with LLC tumor-bearing mice	I.V.	Li et al. (2021)
Natural killer (NK) cells	Chlorin e6	After isolation EVs, incubation	Hepatocellular carcinoma	∼120	*In vitro* tests with HepG2-Luc and CT26 cells and *in vivo* tests with HepG2-Xenograft mice and CT26-Xenograft Tumor mice	S.C.	Zhang et al. (2022)
M1/M2-like macrophages, B16F10 melanoma cancer cells and milk	Zinc Phthalocyanine	After isolation EVs, incubation	Colorectal cancer	100–200	*In vitro* tests with MC38 cells and D1DCs cells and *in vivo* tests with MC38 tumor-bearing mice	I.V.	Huis In 't Veld et al. (2022)
MGC803 cells	Aggregation-induced emission luminogens (AIEgens; TBP-2)	After isolation EVs, incubation	Gastric cancer	50–200	*In vitro* tests in MGC803 cells and *in vivo* tests in MGC803 gastric cancer subcutaneous mice	I.V.	Zhu et al. (2022)
Dendritic cell	AIE-photosensitizer MBPN-TCyP	Before EVs isolation, incubation	Breast cancer and colorectal cancer	131.14 ± 5.25	*In vitro* tests in 4T1 and CT26 cells and *in vivo* tests in 4T1 breast tumor and CT26 colorectal tumor mice	I.V.	Cao et al. (2022)

I.V, intravenous administration; I.T, intratumoral administration; S.C, subcutaneous injection.

### Preclinical testing of photosensitizer loaded EVs

**FIGURE 3:**
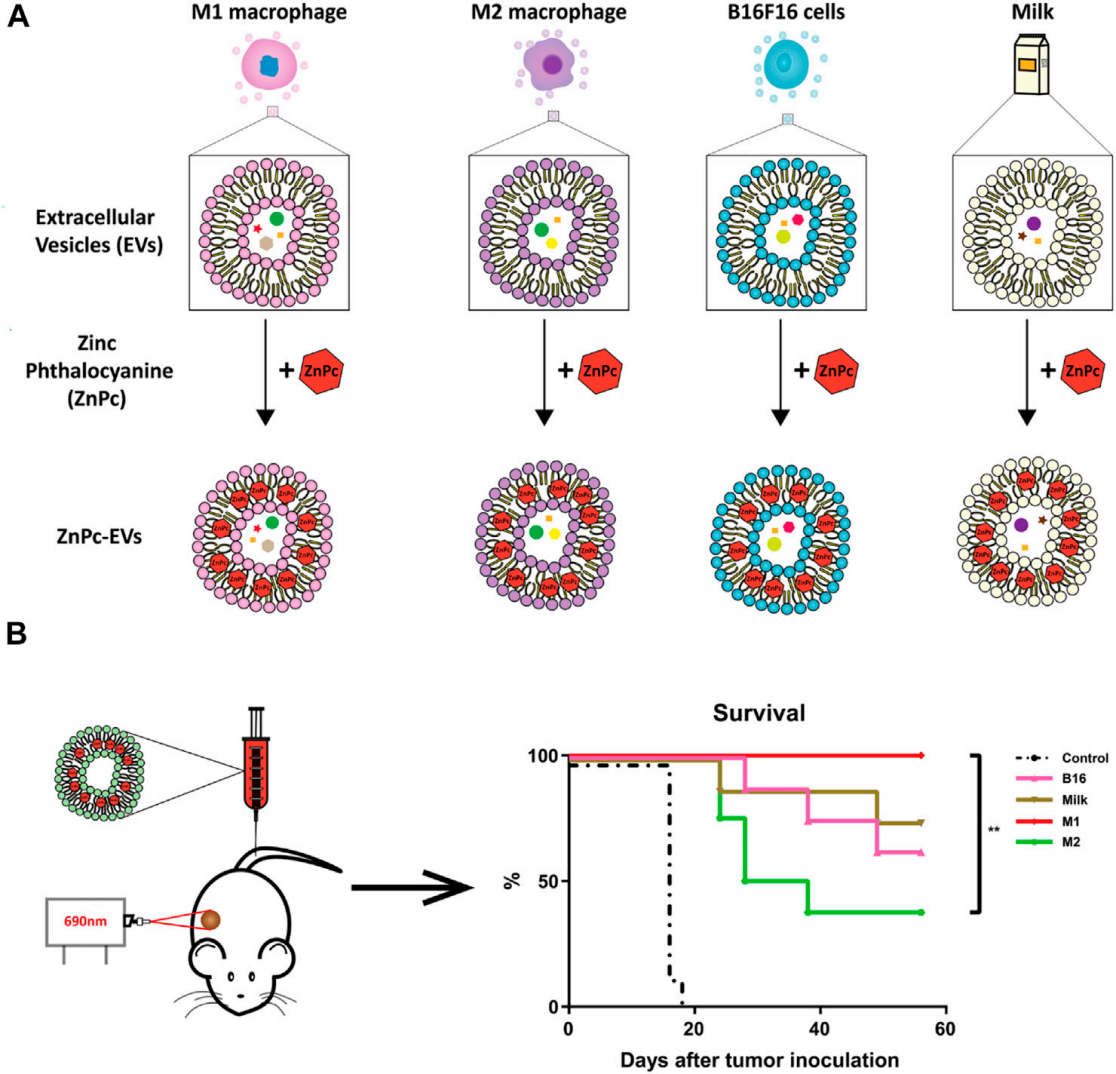
EVs from multiple cell sources as photosensitizer carriers for photodynamic therapy. **(A)** ZnPc was incorporated into EVs derived from immune cells (M1 or M2 like macrophages), cancer cells (B16F10 melanoma cancer cells), or external sources (milk), by a direct incubation strategy. **(B)** Therapeutic efficacy of photosensitizer-based delivery systems in cancer (Huis In 't Veld et al., 2022). Copyright 2022 BMC.

The use of EVs as PS carriers has attracted a great deal of interest among researchers in the past few years. In 2019, the first report documented that chimeric peptide (containing photosensitizer PpIX) engineered exosomes were used to enhance cancer PDT (Cheng et al., 2019). Since then, research on EVs as carriers of PSs has been increasing, accounting for 75% of all preclinical studies during 2021–2022 (Table 3). In these studies, second-generation PSs, such as Ce6, ZnPc, and AlPcS4, were widely used. This may be attributed to their hydrophobic properties, which make them easy to bind to EVs. For instance, Jang et al. (2021) designed the re-assembled Ce6-loaded EVs and evaluated their therapeutic efficiency in pancreatic cancer. Specifically, given the efficient homing and natural immunomodulatory properties of tumor-derived EVs (Batrakova and Kim, 2015), Ce6 was loaded into tumor-derived EVs *via* sonication. As expected, Ce6-loaded EVs retained the ability to selectively target tumor cells and acted as an efficient strategy for combined tumor photodynamic and immunotherapy (Jang et al., 2021). Similarly, Zhang et al. (2022) extracted EVs from natural killer cells (NKs) for loading hydrophobic PS Ce6. Because of the versatile immune regulatory functions of NKs, engineered NK-EVs exhibited remarkable antitumor effects by recruiting multiple types of immune cells and triggering substantial photodynamic therapy effects. These studies suggest that certain intrinsic properties inherited from EV parental cells may enhance the PDT efficacy resulting from PSs delivered by EVs. This concept is supported by a recent study from (Huis In 't Veld et al., 2022). In this study, ZnPc was incorporated into EVs that were isolated from a variety of cellular sources, including M1-like and M2-like macrophages, B16F10 melanoma cancer cells, and external sources like milk (Figure 3A). Interestingly, although all ZnPc-loaded engineered EVs were able to trigger immunogenic cell death and inhibit tumor growth, M1EVs-ZnPc exhibited the best PDT effects (Figure 3B) (Huis In 't Veld et al., 2022). These findings indicate that the anti-tumor PDT outcome is a synergistic effect of ZnPc and its delivery carriers, M1EVs, which inherit the pro-inflammatory property of their parental cells, M1 macrophages.

**FIGURE 4:**
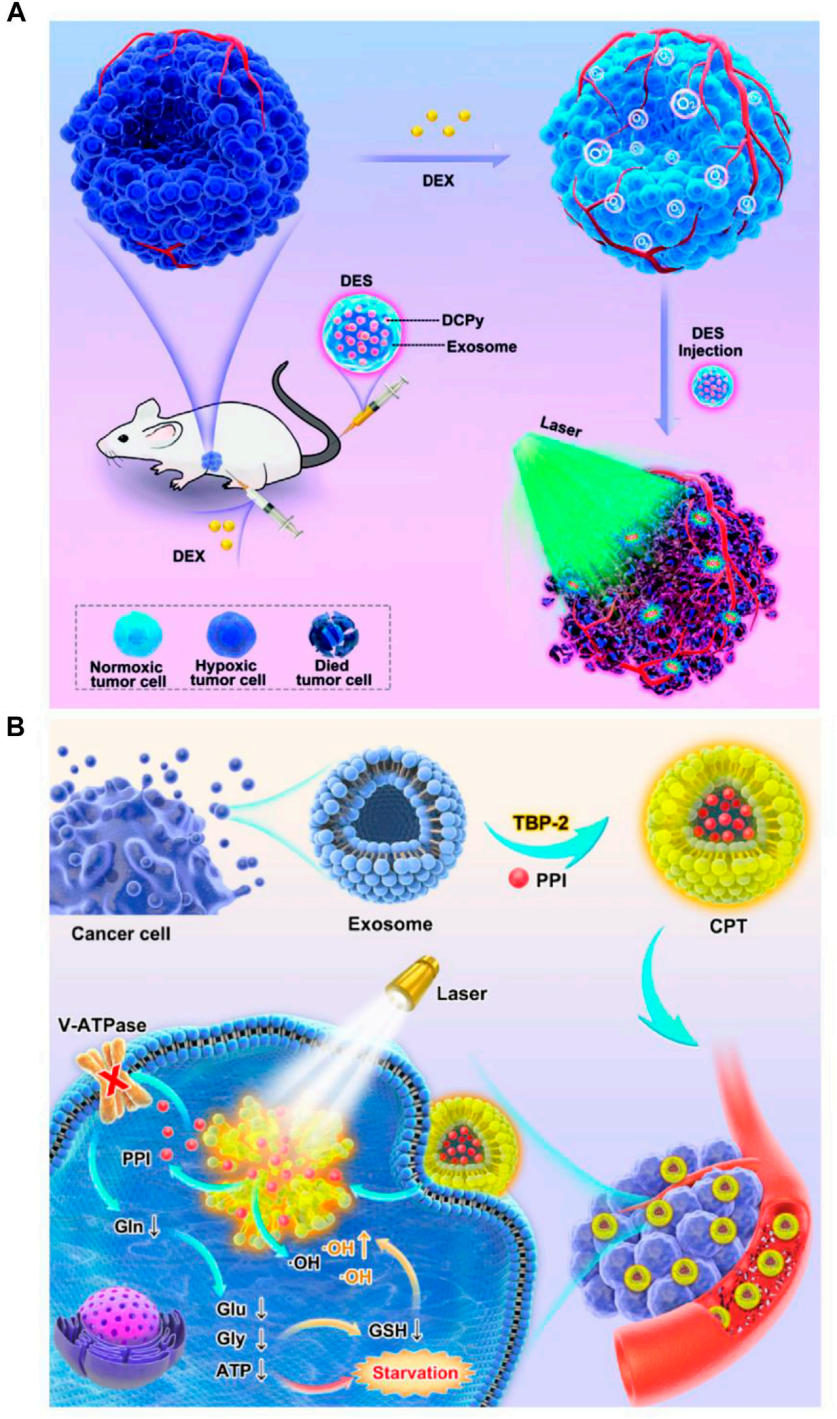
Engineered multi-functional EV delivery platforms for photodynamic therapy. **(A)** Illustration of AIEgens-loaded EV delivery system for promoting efficient tumor penetration and photodynamic therapy (Zhu et al., 2020). **(B)** Schematic illustration of tumor-derived EVs co-delivering AIEgens and proton pump inhibitors for tumor glutamine starvation therapy and enhanced type-I PDT (Zhu et al., 2022). Copyright 2022 German Chemical Society and Elsevier.

In addition to the simple combination between PSs and EVs, multiple therapeutic modalities based on photosensitive EV delivery systems have attracted great attention in enhancing PDT due to their synergistic effects (Pan S. et al., 2020; Li et al., 2021). The first report published by Pan S. et al. (2020) indicated that EV-based nanovehicles were fabricated by urinary EVs loaded with multi-functional PMA/Au-BSA@Ce6 nanoparticles *via* electroporation. Herein, EV-based nanovehicles not only avoid the facile clearance of exogenous nano drug carriers but also enhance targeted PDT due to their deep penetration and excellent retention properties in tumors (Pan S. et al., 2020). This strategy provides a new approach for development of more comprehensive PDT. Moreover, M1 macrophage-derived EVs were used for delivery of upconversion nanoparticles (UC) modified with mesoporous silica, histone deacetylase 1, and suberoylanilide hydroxamic acid and exerted superior anti-tumor effects for the treatment of lung cancer (Li et al., 2021). AIEgens, a type of PSs widely used in PDT (Zhu et al., 2018), have been loaded into lipid-based nanoparticles like PEG-nanoparticles for tumor therapy (Wang et al., 2018a; Sheng et al., 2018). However, poor drug loading and low encapsulation efficiency of AIEgens limit clinical application of these lipid-based nanoparticles in PDT (Qi et al., 2018; Yang Y. et al., 2019). Thus, Zhu and his colleagues designed biomimetic AIEgen/EVs (tumor derived-EVs) hybrid nanovesicles that were able to interact with vascular normalizers, such as glucocorticosteroid dexamethasone (DEX) and proton pump inhibitor (PPI) (Figure 4). These multi-functionalized nanovesicles strongly inhibited glutamine metabolism and enhanced intratumoral ROS production upon laser irradiation, thereby enhancing PDT performance (Zhu et al., 2020; Zhu et al., 2022). Although EVs have been explored to deliver various PSs for PDT, the synergistic effects of the intrinsic properties EVs inherited from their parental cells and the varied loading efficiency of EVs derived from different cell types shall be further defined. In addition, to move to clinical trials, large scale production of EVs or EV mimetics needs to be considered (Ou et al., 2021b). To increase the production yield of EVs for PS delivery, Cao et al. (2022) have developed a straightforward strategy in which dendritic cells are used as a cell reactor to exocytose high-efficient DEVs-mimicking AIE nanoparticles (DEVs-AIE). If this cell reactor approach can be applied for large scale production of EVs from various other cell types, EV delivery-based new generation of PSs may get close to clinical trials.

### Clinical translation of photosensitizers-loaded EVs

To date, five clinical trials have used EVs as drug delivery systems for the treatment of cancer and stroke diseases (Escude Martinez de Castilla et al., 2021), including NCT03384433, NCT03608631, NCT01294072, NCT01854866, and NCT02657460. Among them, there are 2 phase I clinical trials, 1 phase I/II clinical trial, and 2 phase II clinical trials. Given that EV-based PSs delivery systems are still in their early stages of preclinical research, no clinical trials of photosensitizer-loaded EVs for PDT have been reported to date. Despite EV-based drug delivery systems have exhibited certain advantages over conventional nanoparticle systems, their clinical translation remains challenging. This is attributed to the inherent complexity of EVs themselves, such as low yield, poor purity, and high heterogeneity. Compared with purely synthetic nanoparticle production, these inherent challenges in the production of EVs or EV mimetics may be a major limiting factor in the clinical translation advancement (Ou et al., 2021b; Herrmann et al., 2021). Recently, various drug loading and ligand-conjugated target delivery EV platforms have been developed (Elsharkasy et al., 2020; Escude Martinez de Castilla et al., 2021), including 1) the natural EVs released from genetically engineered cells; 2) EVs modified with drugs and surface ligands; 3) hybrid nanovehicles that fuse EVs with other nanoparticles such as liposomes (Goh et al., 2018; Ou et al., 2021a). While these platforms can bring us much closer to clinical trials, the reproducible production procedures are complicated and therefore require additional process control. Nonetheless, with numerous ongoing studies, EV-based PSs delivery systems hold great promise for translation into clinical therapy in the future.

## Conclusions and perspectives

PDT has been explored and shown clinical efficacy for the treatment of cancer, gastrointestinal diseases, cardiovascular disorders, and diabetic ocular disease. However, the therapeutic efficacy of PDT is limited by local and systemic toxicity due to poor targeting of PSs. New generation PSs that are designed by employing surface modifications or nanoparticular delivery systems have enhanced targeting and minimized off-target side effects. However, conventional nanoparticular systems raise certain safety concerns due to their synthetic components. As naturally cell-secreted nanoparticles, EVs have been extensively explored in recent years for drug delivery due to their intrinsic homing property and lower immunogenicity. For delivery of PSs, tumor-derived EVs have shown clear “homing” property to increase accumulation of PSs at the tumor site and enhance antitumor effects (Batrakova and Kim, 2015). On the other hand, those immune cells derived EVs used for PS delivery exhibit remarkable synergistic effects in PDT (Zhu et al., 2019). The lower immunogenicity of EVs in principle would minimize nanoparticle infusion-induced adverse immune responses (Ou et al., 2022), which in turn facilitate development of EV-based PSs. Yet, studies on EVs for PS delivery in PDT remain scarce. The main obstacle is the complexity of EV biogenesis, isolation and purification. For EVs isolated from cell culture medium, cell culture conditions, including cell density and cell passage, strongly influence the EV yield, composition and bioactivity. EVs derived from biofluids such as milk, and edible EVs derived from plant, contain abundant lipoproteins and soluble lipoprotein particles, thereby increasing complexity of EV isolation and purification. Nevertheless, although the clinical translation of EVs for drug delivery is facing various challenges, such as large-scale production and purification, EVs may contribute to the design of a newer generation of PSs for more patient-friendly PDT.

Currently, EVs as PS carriers have been investigated to treat various cancers, surprisingly, not explored yet in any other disease. Given that PDT has been applied to non-cancer diseases, such as gastrointestinal diseases, cardiovascular diseases, and diabetic diseases, it is therefore tempting to speculate that photosensitizers-loaded EVs may enhance PDT efficacy in these diseases as reported in cancer treatment.
